# The impact of perivascular tissue preservation on 5-year patency of saphenous vein composite grafts

**DOI:** 10.1093/icvts/ivae069

**Published:** 2024-04-18

**Authors:** Suk Ho Sohn, Yoonjin Kang, Ji Seong Kim, Jae Woong Choi, Ho Young Hwang

**Affiliations:** Department of Thoracic and Cardiovascular Surgery, Seoul National University Hospital, Seoul National University College of Medicine, Seoul, Republic of Korea; Department of Thoracic and Cardiovascular Surgery, Seoul National University Hospital, Seoul National University College of Medicine, Seoul, Republic of Korea; Department of Thoracic and Cardiovascular Surgery, Seoul National University Hospital, Seoul National University College of Medicine, Seoul, Republic of Korea; Department of Thoracic and Cardiovascular Surgery, Seoul National University Hospital, Seoul National University College of Medicine, Seoul, Republic of Korea; Department of Thoracic and Cardiovascular Surgery, Seoul National University Hospital, Seoul National University College of Medicine, Seoul, Republic of Korea

**Keywords:** Coronary artery bypass grafting, Saphenous vein, Angiography, Outcomes

## Abstract

**OBJECTIVES:**

This retrospective study was conducted to evaluate the impact of saphenous vein (SV) harvesting with versus without perivascular tissue on the 5-year angiographic patency in coronary artery bypass grafting.

**METHODS:**

Among the 944 patients who received coronary artery bypass grafting between 2010 and 2015, 579 patients who received off-pump coronary artery bypass grafting using 1 SV as a Y-composite graft based on the *in situ* left internal thoracic artery were enrolled. SV harvesting was performed using no-touch technique without perivascular tissue (the NoPVT group) in 342 patients and with perivascular tissue (the PVT group) in 237 patients. Follow-up duration was 84.0 months (interquartile range 66.5–105.4). Propensity score matching was performed, and long-term clinical outcomes and angiographic patency were compared.

**RESULTS:**

The average number of distal anastomoses per patient was comparable between the groups, although more SV grafts were anastomosed to left anterior descending territory in the PVT group than in the NoPVT group. Overall survival and cumulative incidence of cardiac death were comparable between the groups, whereas cumulative incidence of target vessel revascularization (1.3% vs 4.3% at 5 year, *P *=* *0.009) and that of major adverse cardiac events (7.3% vs 9.9% at 5 year, *P *=* *0.035) were lower in the PVT group than in the NoPVT group. One-year and 5-year angiographic patency rates of the SV grafts were higher in the PVT group than in the NoPVT group [97.0% vs 91.7% (*P *=* *0.004) and 96.3% vs 89.9% (*P *=* *0.007), respectively].

**CONCLUSIONS:**

SV grafts harvested using no-touch technique with perivascular tissue further improved the 5-year patency of SV composite grafts compared with those without perivascular tissue.

## Graphical Abstract

**Figure:**
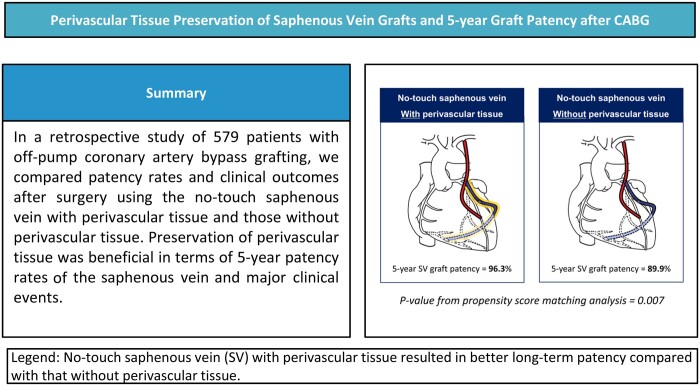

## INTRODUCTION

The internal thoracic artery is considered the 1st graft of choice for coronary artery bypass grafting (CABG) because of its excellent long-term patency and survival benefit [1, 2]. Regarding the 2nd graft of choice, the use of multiple arterial grafting is still limited, and this strategy is performed in only 3.9–34.2% of CABG patients in North America and Europe [3]. In contrast, the saphenous vein (SV) graft, which was introduced more than 50 years ago [4], remains the most commonly used conduit despite the limitations of high failure rates in the short and long term [5–7].

Based on the no-touch (NT) SV harvesting technique, in which the SV is harvested and treated with minimal manipulation and manual intraluminal distension of the SV is avoided [8], a novel NT-SV grafting strategy that harvests the NT-SV with its surrounding perivascular tissue (PVT) was 1st introduced in 1996 [9], and previous studies have reported superior short- and long-term graft patency of novel NT-SV grafts compared with that of conventional SV grafts [10–12]. In addition to the benefits of preventing traumatic manipulation, avoiding manual luminal distension and preserving the vasa vasorum, previous studies have suggested that preserving the PVT might have additional benefits for the NT-SV [13, 14].

Therefore, the present retrospective observational single-centre study was conducted to investigate the impact of PVT preservation during SV harvest on the 5-year angiographic patency of SV grafts.

## MATERIALS AND METHODS

### Patient selection

**Figure 1:**
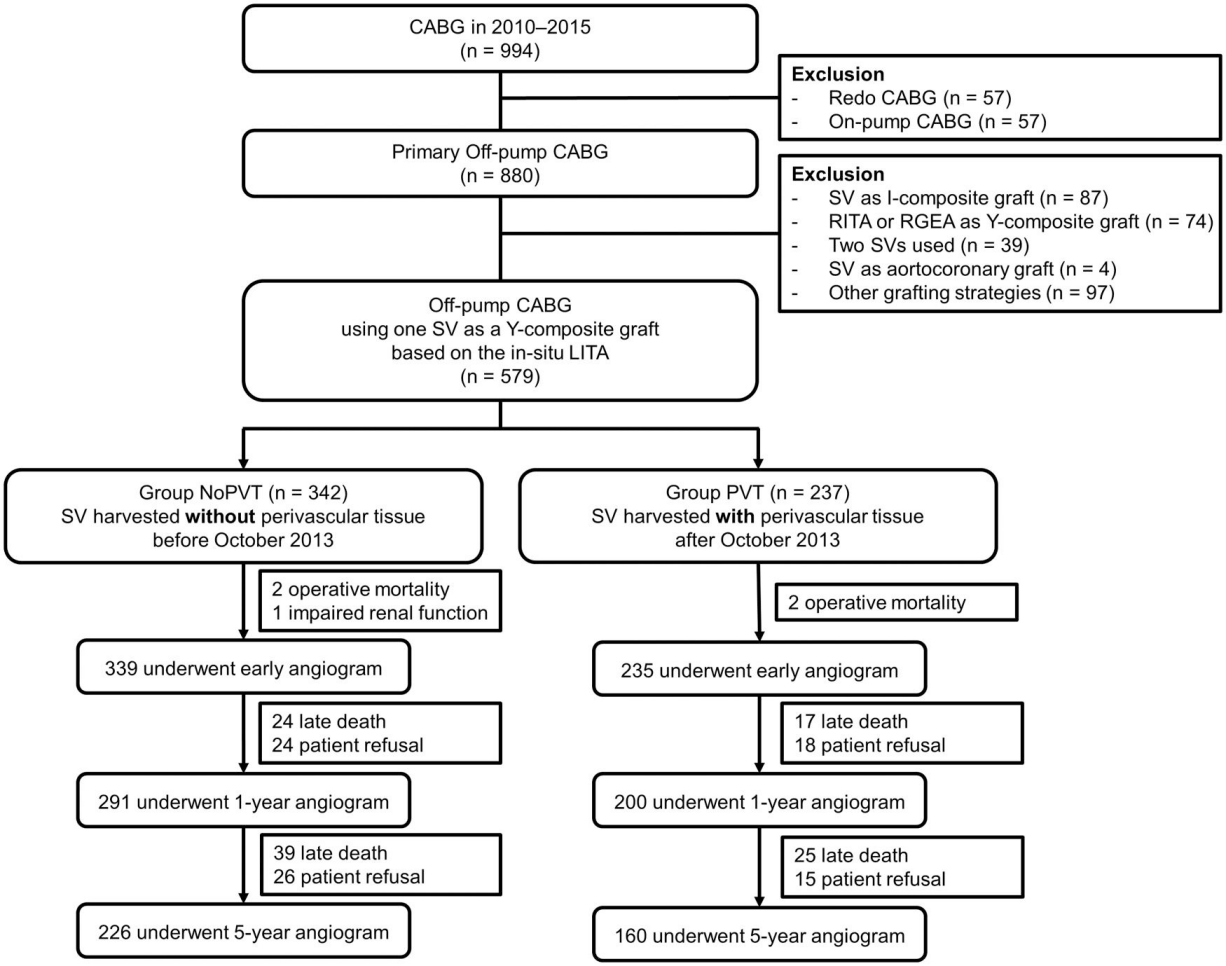
Flow diagram of patient enrolment. CABG: coronary artery bypass grafting; SV: saphenous vein; PVT: perivascular tissue; RGEA: right gastroepiploic artery; RITA: right internal thoracic artery.

The study protocol was reviewed by the Institutional Review Board and approved as a minimal risk retrospective study (approval number H-2201-116-1293), and individual consent was waived. Of 994 patients who received CABG between January 2010 and December 2015 at our institution, primary isolated off-pump CABG was performed in 880 patients. Among these patients, 579 patients who received off-pump CABG using the SV as a Y-composite graft based on *in situ* left internal thoracic artery (LITA) were enrolled in the present study. The SV was harvested using the NT technique with PVT (the PVT group) and without PVT (the NoPVT group) in 237 and 342 patients, respectively (Fig. 1).

### Operative techniques and revascularization strategy

**Figure 2:**
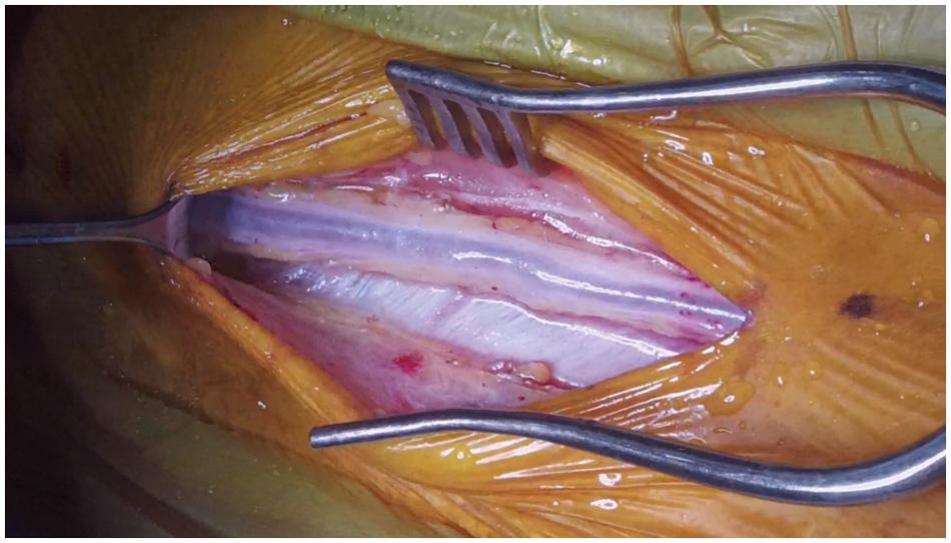
No-touch saphenous vein graft harvest with perivascular tissue.

The operative procedures and surgical strategies of CABG have been illustrated in previous studies [15, 16]. During the study period, anaortic off-pump CABG using Y-composite grafting based on the *in situ* LITA was the preferred revascularization strategy for CABG at our institution to minimize cerebrovascular complications [17]. The SV has been used as the preferred 2nd conduit of choice to construct Y-composite grafts based on our studies showing non-inferiority of the SV compared to arterial conduits [16, 18]. The NT-SV was harvested without PVT until September 2013. Since October 2013, the NT-SV was harvested with a substantial margin of PVT on both sides of the SV and with thin layers of adherent connective tissues anteriorly and posteriorly (Fig. 2).

The NT-SV was harvested preferentially from a lower leg to alleviate the possible size mismatch with native coronary arteries or ITAs, although it resulted in numbness around the lower leg incision in most of the patients. Immediately after the harvest, the reversed SV was anastomosed to the posterior aspect of the mid-LITA in an end-to-side fashion to construct a Y-composite graft. After the construction of the Y-composite graft, the left anterior descending coronary artery territory was revascularized 1st. Then, the left circumflex coronary artery territory was revascularized, followed by right coronary artery territory. A sequential anastomotic technique was also used as needed.

### Evaluation of clinical outcomes

Operative mortality was defined as any mortality within 30 days after surgery or during the same hospitalization. After discharge, all patients visited the outpatient clinic regularly at 3- to 6-month intervals. If the patients had not visited the outpatient clinic as scheduled, their survival and conditions were checked by telephone. For the patients in the loss of follow-ups, their survival data were obtained from the National Health Insurance Service and the Statistics Korea, a central organization for statistics under the Ministry of Strategy and Finance. The clinical follow-up was closed on 31 December 2021. Follow-up data were complete in 100% and 99.5% (576 out of 579 patients) for survival and the other clinical outcomes, respectively, with a median follow-up duration of 84.0 months [interquartile range (IQR) 66.5–105.4].

Cardiac death was defined as any death related to cardiac events, including sudden death during follow-up. Reintervention included both percutaneous coronary intervention and coronary reoperation. Reintervention was further divided into target vessel revascularization (TVR), which was defined as coronary interventions of target vessels that were bypassed during the index operation, and non-TVR, which was defined as coronary interventions for a new lesion outside the target vessels. Major adverse cardiac events included cardiac death, acute myocardial infarction and coronary reintervention.

### Angiographic evaluation of graft patency

Early, 1-year and 5-year follow-up coronary angiograms were performed based on the patient’s consent, regardless of angina symptoms. Patients who died, refused angiographic evaluation or had renal function impairment but were not dependent on renal replacement therapy were excluded from angiographic follow-up. Early postoperative [1 day (IQR 1–1)] graft evaluation was performed in 99.1% (574 out of 579) of the study patients with conventional angiography. One-year [12.5 months (IQR 12.1–13.3)] graft evaluation was performed in 491 patients (84.8%) using conventional angiography and multidetector computed tomography (MDCT) in 274 and 217 patients, respectively. Five-year [60.5 months (IQR 59.9–62.1)] graft evaluation was performed in 386 patients (66.7%) with conventional angiography and MDCT in 85 and 301 patients, respectively (Supplementary Material, Table S1).

### Statistical analysis

Statistical analysis was performed using IBM SPSS version 25.0 (IBM Corp., Armonk, NY, USA) and SAS version 9.4 (SAS Institute Inc., Cary, NC, USA). Continuous variables are presented as the mean ± standard deviation or median with interquartile range, and categorical variables are presented as the number and proportion. Propensity score-matched analysis was performed to adjust the differences in preoperative characteristics. Eighteen variables in baseline characteristics were selected for the propensity score-matched analysis including age, sex, smoking, body mass index >25.0 kg/m^2^, hypertension, diabetes mellitus, dyslipidaemia, history of stroke, chronic kidney disease, chronic obstructive pulmonary disease, atrial fibrillation, peripheral vascular disease, previous percutaneous coronary intervention, left ventricular ejection fraction <0.35, preoperative diagnosis, 3-vessel disease, left main disease and emergency operation. After calculating the propensity scores, 232 pairs were matched using a nearest neighbourhood within a calliper width of 0.1 in propensity scores and with a ratio of 1:1. The balance of covariates between the groups was evaluated with the standardized mean difference. A standardized mean difference of <0.100 was considered as a negligible difference between the groups.

In the unmatched population, the 2 groups were compared using the chi-square test or Fisher’s exact test for categorical variables and Student’s *t*-test or Wilcoxon rank sum test for continuous variables. In the matched population, McNemar tests were performed for categorical variables, and Wilcoxon signed rank sum tests or paired t tests were performed for continuous variables. Comparison of the graft patency rates between the 2 groups was performed using logistic regression with generalized estimating equations to account for the clustering of multiple measurements per subject.

For longitudinal data analyses, overall survival was estimated using the Kaplan–Meier method and compared with the log-rank test. Cardiac death, TVR, reintervention and major adverse cardiac events were analysed with cumulative incidence curves. Noncardiac death was considered a competing event for cardiac death, and all-cause death was considered a competing event for the other outcomes. Comparison of cumulative incidence curves between the 2 groups was performed using a subdistribution hazards model [19]. A *P* value of <0.050 was considered to indicate statistical significance.

## RESULTS

### Patient characteristics

The mean age was 67.0 ± 9.7 years, and 24.2% (140 out of 579) of the patients were female. There were no differences in demographic data or preoperative risk factors between the 2 groups, except for a higher proportion of patients with a history of percutaneous coronary intervention in the PVT group than in the NoPVT group (19.8% vs 13.5%, *P *=* *0.040). After propensity score matching, the differences in patient characteristics between the groups significantly reduced (Table 1).

**Table 1:** Preoperative characteristics and risk factors for the study patients

	All study patients				Propensity-matched patients			
Variables	Group PVT (*n* = 237)	Group NoPVT (*n* = 342)	SMD	*P*	Group PVT (*n* = 232)	Group NoPVT (*n* = 232)	SMD	*P*
Age (years)	67.5 ± 9.4	66.7 ± 9.9	0.073	0.588	67.4 ± 9.5	66.8 ± 10.4	0.050	0.865
Sex (female), *n* (%)	60 (25.3)	80 (23.4)	0.045	0.595	60 (25.9)	57 (24.6)	0.030	0.824
EuroSCORE II	1.2 (0.9–2.2)	1.4 (0.9–2.5)	–0.044	0.176	1.2 (0.9–2.2)	1.5 (0.9–2.5)	–0.038	0.292
Risk factors, *n* (%)								
Smoking	75 (31.6)	133 (38.9)	–0.152	0.074	73 (31.5)	84 (36.2)	–0.100	0.284
Body mass index >25.0 kg/m^2^	86 (36.3)	143 (41.8)	–0.114	0.181	85 (36.6)	91 (39.2)	–0.054	0.624
Hypertension	169 (71.3)	241 (70.5)	0.019	0.827	166 (71.6)	164 (70.7)	0.019	0.918
Diabetes mellitus	113 (47.7)	160 (46.8)	0.018	0.832	110 (47.4)	104 (44.8)	0.052	0.643
Dyslipidaemia	85 (35.9)	123 (36.0)	–0.002	0.980	84 (36.2)	90 (38.8)	–0.053	0.634
History of stroke	31 (13.1)	49 (14.3)	–0.036	0.669	31 (13.4)	33 (14.2)	–0.025	0.896
Chronic kidney disease	59 (24.9)	79 (22.2)	0.063	0.455	57 (24.6)	56 (24.1)	0.010	>0.999
Chronic obstructive pulmonary disease	11 (4.6)	8 (2.3)	0.126	0.126	8 (3.4)	8 (3.4)	<0.001	>0.999
Atrial fibrillation	5 (2.1)	8 (2.3)	–0.016	0.855	5 (2.2)	6 (2.6)	–0.028	>0.999
Peripheral vascular disease	12 (5.1)	16 (4.7)	0.018	0.832	12 (5.2)	12 (5.2)	<0.001	>0.999
Previous percutaneous coronary intervention	47 (19.8)	46 (13.5)	0.172	0.040	44 (19.0)	44 (19.0)	<0.001	>0.999
Left ventricular ejection fraction <0.35	27 (11.4)	54 (15.8)	–0.129	0.134	26 (11.2)	25 (10.8)	0.014	>0.999
Preoperative diagnosis, *n* (%)								
Stable angina	86 (36.3)	112 (32.7)	0.075	0.377	83 (35.8)	83 (35.8)	<0.001	>0.999
Acute coronary syndrome	151 (63.7)	230 (67.3)	–0.075	0.377	149 (64.2)	149 (64.2)	<0.001	>0.999
Three-vessel disease, *n* (%)	188 (79.3)	277 (81.0)	–0.042	0.619	185 (79.7)	184 (79.3)	0.011	>0.999
Left main disease, *n* (%)	107 (45.1)	132 (38.6)	0.133	0.115	102 (44.0)	102 (44.0)	<0.001	>0.999
Emergency operation, *n* (%)	4 (1.7)	6 (1.8)	–0.005	>0.999	4 (1.7)	4 (1.7)	<0.001	>0.999

Continuous variables are presented as the average ± standard deviation for normally distributed variables, and median with interquartile range for non-normally distributed variables.

PVT: perivascular tissue; SMD: standardized mean difference.

### Operative data

The number of distal anastomoses per patient ranged from 2 to 6, and was comparable between the groups. The number of distal anastomoses per LITA was smaller in the PVT group than in the NoPVT group, whereas that per SV was greater in the PVT group than in the NoPVT group. These findings regarding the numbers of distal anastomoses remained consistent after propensity score matching (Table 2).

**Table 2:** Comparison of the number of distal anastomoses between the groups

	All study patients			Propensity-matched patients		
Variables	Group PVT (*n* = 237)	Group NoPVT (*n* = 342)	*P*	Group PVT (*n* = 232)	Group NoPVT (*n* = 232)	*P*
Number of distal anastomosis per patient						
2	14 (5.9%)	23 (6.7%)	0.692	14 (6.0%)	17 (7.3%)	0.577
3	80 (33.8%)	113 (33.0%)	0.858	78 (33.6%)	75 (32.3%)	0.767
4	100 (42.2%)	159 (46.5%)	0.307	98 (42.2%)	104 (44.8%)	0.574
5	40 (16.9%)	44 (12.9%)	0.178	39 (16.8%)	33 (14.2%)	0.442
6	3 (1.3%)	3 (0.9%)	0.693	3 (1.3%)	3 (1.3%)	>0.999
Number of distal anastomosis per LITA						
1	226 (95.4%)	278 (81.3%)	<0.001	221 (95.3%)	194 (83.6%)	<0.001
2	11 (4.6%)	62 (18.1%)	<0.001	11 (4.7%)	37 (15.9%)	<0.001
3	0 (0.0%)	2 (0.6%)	0.516	0 (0.0%)	1 (0.4%)	>0.001
Number of distal anastomosis per SV						
1	15 (6.3%)	30 (8.8%)	0.280	15 (6.5%)	21 (9.1%)	0.298
2	82 (34.6%)	136 (39.8%)	0.207	80 (34.5%)	86 (37.1%)	0.561
3	103 (43.5%)	155 (45.3%)	0.658	101 (43.5%)	106 (45.7%)	0.641
4	35 (14.8%)	21 (6.1%)	<0.001	34 (14.7%)	19 (8.2%)	0.029
5	2 (0.8%)	0 (0.0%)	0.167	2 (0.9%)	0 (0.0%)	0.499
Anastomosed to LAD territory						
0	80 (33.8%)	173 (50.6%)	<0.001	80 (34.5%)	114 (49.1%)	0.001
1	152 (64.1%)	169 (49.4%)	<0.001	148 (63.8%)	118 (50.9%)	0.005
2	5 (2.1%)	0 (0.0%)	0.011	4 (1.7%)	0 (0.0%)	0.123
Anastomosed to LCx territory						
0	23 (9.7%)	26 (7.6%)	0.371	22 (9.5%)	16 (6.9%)	0.310
1	154 (65.0%)	236 (69.0%)	0.310	150 (64.7%)	156 (67.2%)	0.557
2	58 (24.5%)	79 (23.1%)	0.702	58 (25.0%)	59 (25.4%)	0.915
3	2 (0.8%)	1 (0.3%)	0.571	2 (0.9%)	1 (0.4%)	>0.999
Anastomosed to RCA territory						
0	59 (24.9%)	90 (26.3%)	0.700	57 (24.6%)	66 (28.4%)	0.344
1	156 (65.8%)	219 (64.0%)	0.658	154 (66.4%)	140 (60.3%)	0.177
2	22 (9.3%)	33 (9.6%)	0.882	21 (9.1%)	26 (11.2%)	0.442

Continuous variables are presented as median with interquartile range for non-normally distributed variables.

LAD: left anterior descending; LCx: left circumflex; LITA: left internal thoracic artery; PVT: perivascular tissue; RCA: right coronary artery; SV: saphenous vein.

### Early and long-term clinical outcomes

Operative mortality was 0.9% (5 out of 579 patients) without an inter-group difference. There were no significant differences in the occurrence rates of postoperative complications between the 2 groups both in the entire population and in the matched population, except for the higher incidence of SV harvest site complications in the PVT group (Table 3).

**Table 3:** Comparison of early clinical outcomes between the groups

	All study patients			Propensity-matched patients		
Variables	Group PVT (*n* = 237)	Group NoPVT (*n* = 342)	*P*	Group PVT (*n* = 232)	Group NoPVT (*n* = 232)	*P*
Operative mortality, *n* (%)	3 (1.3)	2 (0.6)	0.404	3 (1.3)	2 (0.9)	>0.999
Postoperative complications, *n* (%)						
Postoperative atrial fibrillation	88 (37.1)	113 (33.0)	0.309	86 (37.1)	76 (32.8)	0.373
Acute kidney injury	14 (5.9)	26 (7.6)	0.429	14 (6.0)	20 (8.6)	0.327
Respiratory complications	14 (5.9)	20 (5.8)	0.976	14 (6.0)	13 (5.6)	>0.999
Delirium	12 (5.1)	11 (3.2)	0.263	12 (5.2)	5 (2.2)	0.143
Bleeding reoperation	5 (2.1)	10 (2.9)	0.606	4 (1.7)	8 (3.4)	0.344
Stroke	1 (0.4)	2 (0.6)	>0.999	1 (0.4)	2 (0.9)	>0.999
Mediastinitis	1 (0.4)	2 (0.6)	>0.999	1 (0.4)	1 (0.4)	>0.999
SV harvest site complications	8 (3.4)	2 (0.6)	0.019	8 (3.4)	2 (0.9)	0.109

PVT: perivascular tissue; SV: saphenous vein.

**Figure 3:**
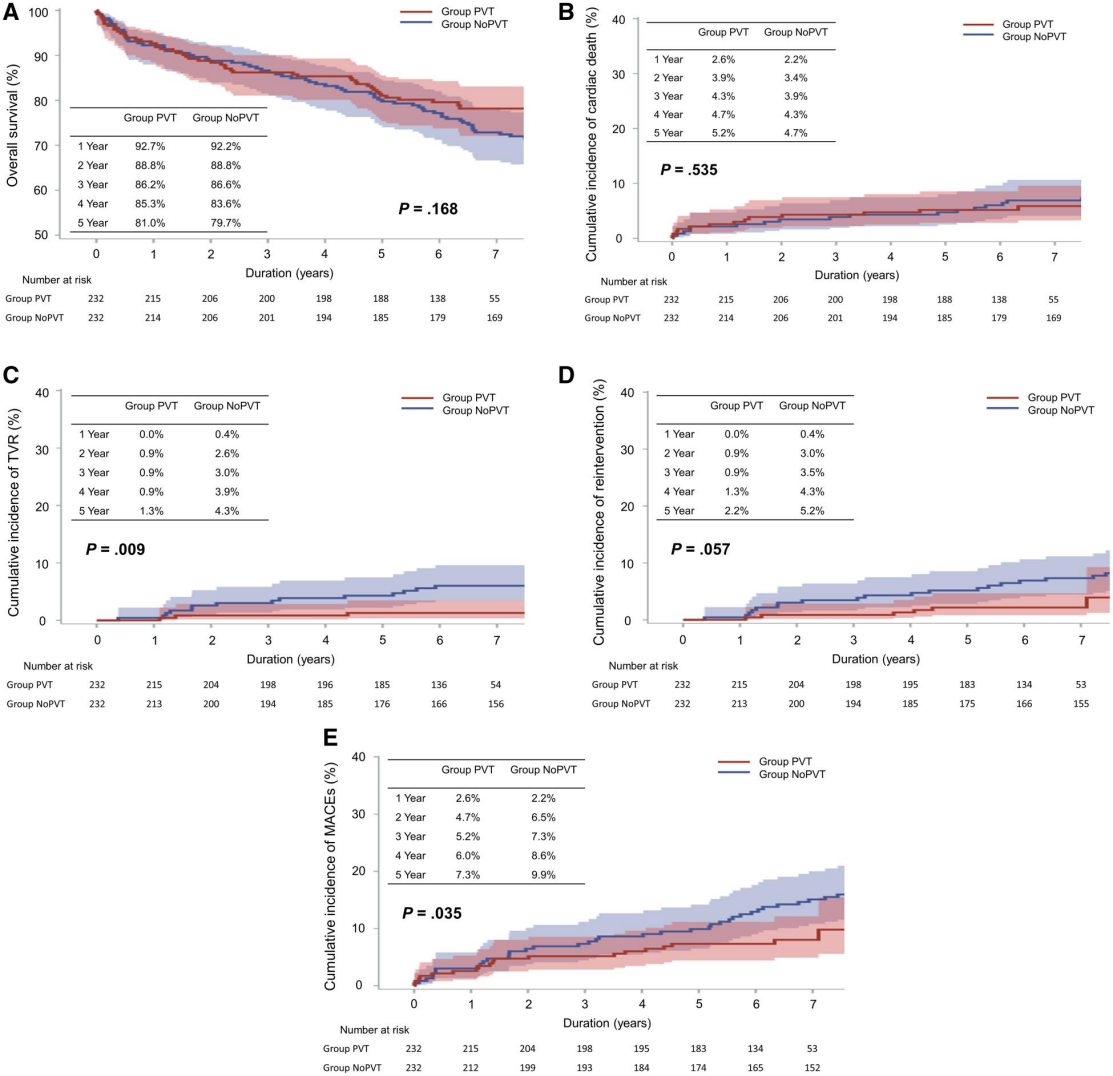
Comparison of (**A**) overall survival, (**B**) cumulative incidence of cardiac death, (**C**) cumulative incidence of target vessel revascularization (TVR), (**D**) cumulative incidence of reintervention and (**E**) cumulative incidence of major adverse cardiac events (MACEs) between the groups in the propensity score-matched population. MACE: major adverse cardiac event; NoPVT: without PVT; PVT: perivascular tissue; TVR: target vessel revascularization.

In the entire population, 5-year overall survival rates (PVT vs NoPVT groups = 81.0% vs 81.0%, *P *=* *0.223) and cumulative incidences of cardiac death (5.5% vs 4.4%, *P *=* *0.936) were not significantly different between the 2 groups, whereas the 5-year cumulative incidence of TVR was lower in the PVT group than in the NoPVT group (1.3% vs 3.2%, *P *=* *0.027) (Supplementary Material, Fig. S1). In the matched population, 5-year overall survival rates and cumulative incidences of cardiac death consistently demonstrated no significant differences between the groups. However, the cumulative incidence of TVR (1.3% vs 4.3% at 5 year, *P *=* *0.009) and that of major adverse cardiac events (7.3% vs 9.9% at 5 year, *P *=* *0.035) were significantly lower in the PVT group than in the NoPVT group. The cumulative incidence of reintervention was also lower in the PVT group with a marginal significance (2.2% vs 5.2% at 5 year, *P *=* *0.057) (Fig. 3).

### Angiographic graft patency

Early, 1-year and 5-year postoperative overall graft patency rates in the PVT versus NoPVT groups were 99.2% vs 97.9%, 97.6% vs 93.3% and 96.5% vs 92.2% in the matched cohort, respectively. All the obstructions occurred in distal anastomosis or distal graft, and no obstruction occurred at the level of Y-graft. There were no significant differences in graft patency rates of LITA between the 2 groups [early, 1 year and 5 years = 99.6% vs 99.6% (*P *=* *0.941), 99.0% vs 97.0% (*P *=* *0.154) and 97.0% vs 97.2% (*P *=* *0.959), respectively], whereas patency rates of SV were significantly higher in the PVT group than in the NoPVT group at early, 1-year and 5-year angiograms (99.0% vs 97.1%, 97.0% vs 91.7% and 96.3% vs 89.9%, respectively) (Table 4). When the graft patency rates of the SV were analysed based on the coronary artery territory, they were highest when anastomosed to the left anterior descending coronary artery territory and lowest when anastomosed to the right coronary artery territory in both groups and in all time periods before and after matching. The patency rates of the SV to the right coronary artery territory showed significant differences between the 2 groups at 1 and 5 years [94.7% (161 of 170 distal anastomoses) vs 83.4% (141 of 169 distal anastomoses), *P *=* *0.003 and 93.3% (126 of 135 distal anastomoses) vs 82.6% (100 of 121 distal anastomoses), *P *=* *0.013, respectively], whereas there were no significant differences in the patency rates of the SV to the left anterior descending coronary artery territory in all time periods (Table 5).

**Table 4:** Comparison of early, 1-year and 5-year angiographic patency rates of all anastomoses between the groups

	All study patients			Propensity-matched patients		
Variables	Group PVT (*n* = 237)	Group NoPVT (*n* = 342)	*P*	Group PVT (*n* = 232)	Group NoPVT (*n* = 232)	*P*
Early angiography	(*n* = 235)	(*n* = 339)		(*n* = 230)	(*n* = 229)	
Overall	99.1% (811/818)	97.9% (1221/1247)	0.021	99.2% (854/861)	97.9% (828/846)	0.029
LITA	99.6% (245/246)	99.3% (402/405)	0.602	99.6% (240/241)	99.6% (267/268)	0.941
SV	99.1% (628/634)	97.3% (819/842)	0.018	99.0% (614/620)	97.1% (561/578)	0.017
One-year angiography	(*n* = 200)	(*n* = 291)		(*n* = 197)	(*n* = 203)	
Overall	97.6% (735/753)	94.0% (1009/1073)	0.003	97.6% (723/741)	93.3% (701/751)	0.002
LITA	99.0% (208/210)	97.7% (335/343)	0.253	99.0% (205/207)	97.0% (227/234)	0.154
SV	97.1% (527/543)	92.3% (675/731)	0.006	97.0% (518/534)	91.7% (475/518)	0.004
Five-year angiography	(*n* = 160)	(*n* = 226)		(*n* = 159)	(*n* = 152)	
Overall	96.6% (588/609)	93.3% (774/830)	0.022	96.5% (581/602)	92.2% (519/563)	0.010
LITA	97.1% (165/170)	97.0% (260/268)	0.962	97.0% (164/169)	97.2% (171/176)	0.959
SV	96.4% (423/439)	91.5% (514/562)	0.020	96.3% (417/433)	89.9% (348/387)	0.007

LITA, left internal thoracic artery; PVT, perivascular tissue; SV, saphenous vein.

**Table 5:** Comparison of early, 1-year and 5-year angiographic patency rates of the anastomoses with saphenous vein grafts between the groups

	All study patients			Propensity-matched patients		
Variables	Group PVT (*n* = 237)	Group NoPVT (*n* = 342)	*P*	Group PVT (*n* = 232)	Group NoPVT (*n* = 232)	*P*
Early angiography	(*n* = 235)	(*n* = 339)		(*n* = 230)	(*n* = 229)	
Overall SV anastomoses	99.1% (628/634)	97.3% (819/842)	0.018	99.0% (614/620)	97.1% (561/578)	0.017
Anastomosed to LAD territory	100.0% (161/161)	100.0% (167/167)	–	100.0% (155/155)	100.0% (116/116)	–
Anastomosed to LCx territory	99.6% (272/273)	98.2% (385/392)	0.136	99.6% (268/269)	98.2% (267/272)	0.142
Anastomosed to RCA territory	97.5% (195/200)	94.3% (267/283)	0.105	97.4% (191/196)	93.7% (178/190)	0.086
One-year angiography	(*n* = 200)	(*n* = 291)		(*n* = 197)	(*n* = 203)	
Overall SV anastomoses	97.1% (527/543)	92.3% (675/731)	0.006	97.0% (518/534)	91.7% (475/518)	0.004
Anastomosed to LAD territory	98.5% (132/134)	97.9% (140/143)	0.708	98.5% (129/131)	98.1% (103/105)	0.825
Anastomosed to LCx territory	97.9% (231/236)	94.8% (328/346)	0.091	97.9% (228/233)	94.7% (231/244)	0.104
Anastomosed to RCA territory	94.8% (164/173)	85.5% (207/242)	0.006	94.7% (161/170)	83.4% (141/169)	0.003
Five-year angiography	(*n* = 160)	(*n *= 226)		(*n* = 159)	(*n* = 152)	
Overall SV anastomoses	96.4% (423/439)	91.5% (514/562)	0.020	96.3% (417/433)	89.9% (348/387)	0.007
Anastomosed to LAD territory	99.1% (108/109)	96.5% (109/113)	0.222	99.1% (105/106)	96.4% (80/83)	0.239
Anastomosed to LCx territory	96.9% (187/193)	93.0% (251/270)	0.103	96.9% (186/192)	91.8% (168/183)	0.063
Anastomosed to RCA territory	93.4% (128/137)	86.0% (154/179)	0.044	93.3% (126/135)	82.6% (100/121)	0.013

LAD: left anterior descending; LCX: left circumflex; PVT: perivascular tissue; RCA: right coronary artery; SV: saphenous vein.

## DISCUSSION

The present study demonstrated that preservation of PVT in NT-SV harvesting might be beneficial in terms of 5-year angiographic patency and cumulative incidence of TVR after CABG.

The concept of preserving the wall characteristics of SV grafts by the NT technique, which was inspired by the poor patency rates of SV grafts after CABG, was introduced in the early period of CABG, but it was not popularized [8]. A novel NT-SV grafting strategy was then introduced in 1996 [9]. Following the harvest methods of NT-SV as originally described, the novel strategy included preservation of the surrounding PVT. Although recent guidelines recommend NT-SV as Class IIa when an open technique is used [20], data regarding the benefits of NT-SV with PVT in CABG are limited because most studies have been reported from Souza and his colleagues, who introduced this technique [10–12, 21, 22].

In addition to the benefit of the NT technique that prevents the SV wall structures, particularly endothelial integrity, from mechanical damage during surgery, preserving the PVT in the novel NT technique has several theoretical advantages. These included (i) saving the vasa vasorum that prevents the SV from transmural ischaemic damage and (ii) buffering against pulsatile stress of the arterial flow that reduces neointimal and medial thickening [23–26]. With this theoretical background, a previous randomized controlled trial enrolled 52 patients in each NT-SV and control group. That study showed that aortocoronary NT-SV with PVT resulted in favourable patency rates of 95.4%, 90% and 83% at 1.5, 8.5 and 16 years after CABG, respectively, although 16-year patency was evaluated in only 27 patients with 75 distal anastomoses [10, 11, 21]. Another randomized trial that enrolled 250 patients from multiple institutions demonstrated that the composite rate of severe stenosis or occlusion at 12 months after CABG was 7.8% (8 of 102 grafts) in the NT-SV group [27].

In the present study, the NT-SV grafts were all used as composite grafts based on *in situ* LITA. Study results showed that 1-year and 5-year patency of the NT-SV grafts without PVT were 92.3% and 91.5%, respectively, which might be lower than the former randomized controlled trial but comparable to the latter randomized controlled trial. The patency rates of the NT-SV in the PVT group were significantly higher than those in the NoPVT group and were comparable with those in previous studies using NT-SV with PVT.

In addition to the theoretical advantages described above, there has been growing evidence regarding the impact of the PVT on the NT-SV. Previous studies have suggested that adipose tissue in the PVT interacts with vascular smooth muscle cells and modulates vascular tone as well as externally supports vascular structures [28, 29]. Another study revealed that it is a major source of nitric oxide, which plays crucial roles in suppressing atherosclerosis [14]. In that study, the authors analysed residual SVs obtained during CABG and demonstrated that the PVT expressed high levels of enzymes related to nitric oxide synthesis, such as argininosuccinate synthase 1 and endothelial nitric oxide synthase.

The present study confirmed that the PVT of the NT-SV is beneficial for patency of the NT-SV grafts up to 5 years after surgery. These improved patencies might result in favourable clinical outcomes in the PVT group, although those outcomes were not significantly different from those of the NoPVT group, except for the cumulative incidence of TVR.

### Study limitations

There are several limitations to the present study that must be recognized. First, this study was a single institutional study and was not performed in a prospective randomized manner, although all consecutive patients who met the inclusion criteria were enrolled and propensity score matching was performed. Second, 1-year and 5-year graft evaluations were not performed in all the study patients, and graft patency rates might be overestimated. Third, there was a discrepancy in the modality, conventional angiogram versus MDCT, to evaluate the graft patency. Although the diagnostic efficacy of MDCT has been well demonstrated as excellent [30], this discrepancy could affect the results. Fourth, patients were recruited to this study at different time points with no overlap. Although propensity score matching was performed to overcome retrospective nature of the present study, other changes to surgical practice may have influenced the study results. Fifth, the effect of early technical failure to the long-term patency should also be considered in the interpretation of the results because the difference in graft patency existed at early angiographic evaluation.

## CONCLUSIONS

Preserving the PVT during NT-SV harvest might improve graft patency rates of the SV up to 5 years after CABG.

## Supplementary Material

ivae069_Supplementary_Data

## Data Availability

The data underlying this article will be shared on reasonable request to the corresponding author.

## Notes

Presented at the AATS 103rd Annual Meeting in Los Angeles, May 7th 2023, as a poster.

## ABBREVIATIONS

CABG: Coronary artery bypass grafting
IQR: Interquartile range
LITA: Left internal thoracic artery
MDCT: Multidetector computed tomography
NoPVT: Without perivascular tissue
NT: No-touch
PVT: Perivascular tissue
SV: Saphenous vein
TVR: Target vessel revascularization
