# Supplementary material for: The impact of perivascular tissue preservation on 5-year patency of saphenous vein composite grafts
Source: Interdiscip Cardiovasc Thorac Surg. 2024 Apr 18;38(5):ivae069. doi: 10.1093/icvts/ivae069 (PMC11076921; doi:10.1093/icvts/ivae069)
Supplement: ivae069_Supplementary_Data [file ivae069_supplementary_data.docx]

**Supplementary material**

**Figure S1:** Comparison of (A) overall survival, (B) cumulative incidence of cardiac death, (C) cumulative incidence of target vessel revascularization (TVR), (D) cumulative incidence of reintervention, and (E) cumulative incidence of major adverse cardiac events (MACEs) between the groups in the entire population.

(A)


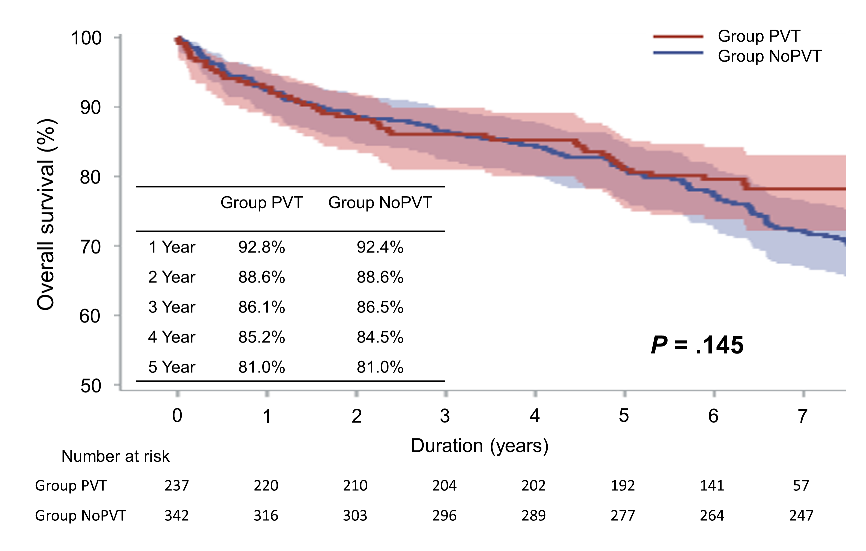


(B)


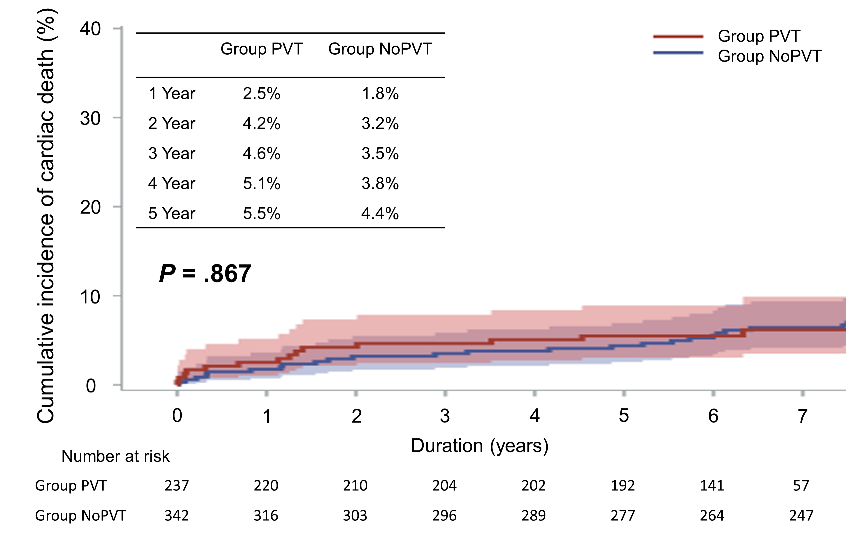


(C)


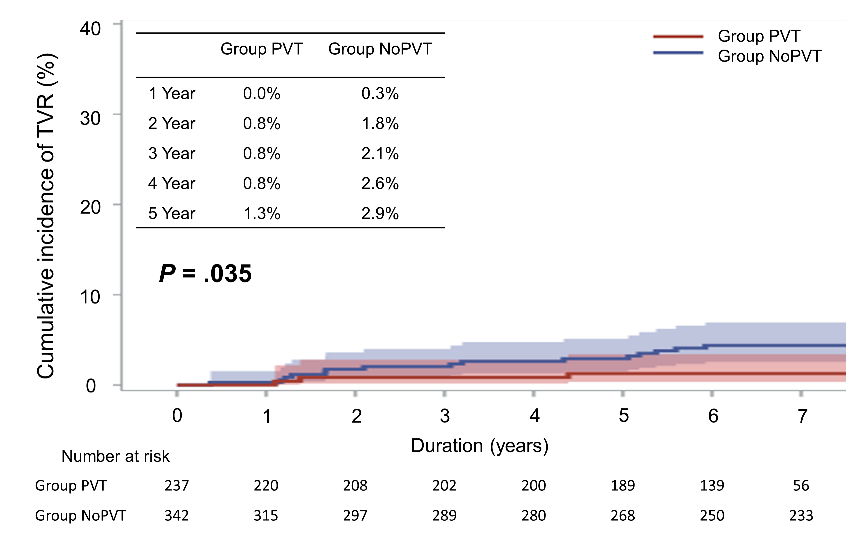


(D)


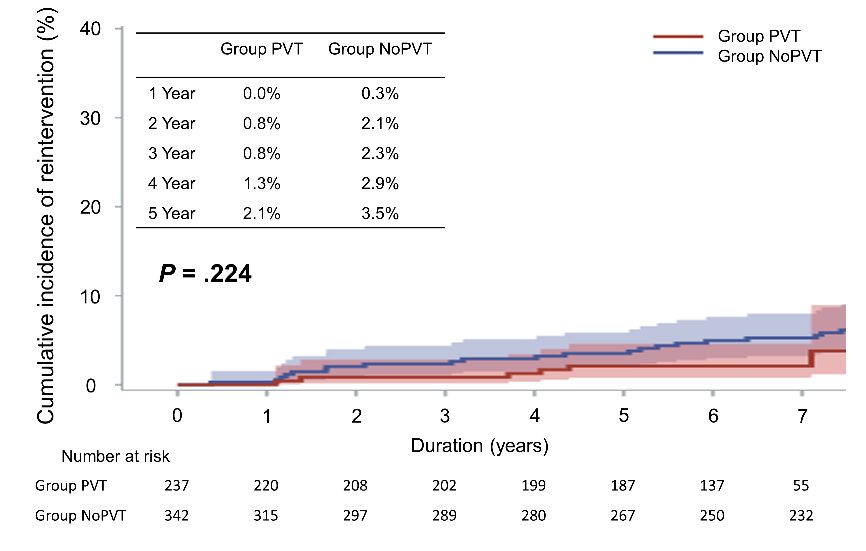


(E)


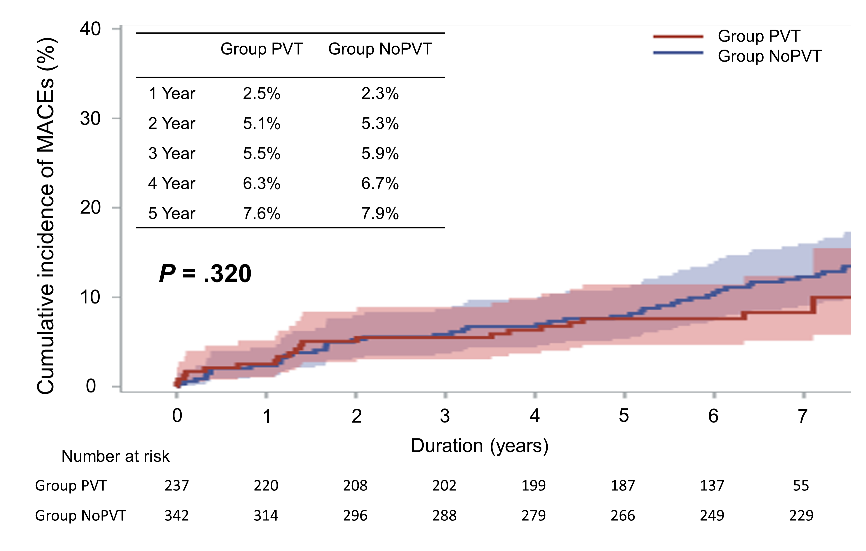


**Table S1:** Proportion of CT angiography in early, 1-year, and 5-year angiography

| Variables | All Study Patients | |  | Propensity-Matched Patients | |
| --- | --- | --- | --- | --- | --- |
|  | Group PVT  (n = 237) | Group NoPVT  (n = 342) |  | Group PVT  (n = 232) | Group NoPVT  (n = 232) |
| Early angiography | (n = 235) | (n = 339) |  | (n = 230) | (n = 229) |
| Conventional angiography | 235 (100.0%) | 339 (100.0%) |  | 230 (100.0%) | 229 (100.0%) |
| CT angiography | 0 (0.0%) | 0 (0.0%) |  | 0 (0.0%) | 0 (0.0%) |
| 1-year angiography | (n = 200) | (n = 291) |  | (n = 197) | (n = 203) |
| Conventional angiography | 70 (35.0%) | 204 (70.1%) |  | 68 (34.5%) | 203 (70.0%) |
| CT angiography | 130 (65.0%) | 87 (29.9%) |  | 129 (65.5%) | 61 (30.0%) |
| 5-year angiography | (n = 160) | (n = 226) |  | (n = 159) | (n = 152) |
| Conventional angiography | 9 (5.6%) | 76 (33.6%) |  | 11 (6.9%) | 51 (33.6%) |
| CT angiography | 151 (94.4%) | 150 (66.4%) |  | 148 (93.1%) | 101 (66.4%) |

CT, computed tomography; PVT, perivascular tissue.
